# Developing the amazing photocatalyst of ZnAg_2_GeSe_4_, ZnAg_2_Ge_0.93_Fe_0.07_Se_4_ and ZnAg_2_Ge_0.86_Fe_0.14_Se_4_ through the computational explorations by four DFT functionals

**DOI:** 10.1016/j.heliyon.2021.e07467

**Published:** 2021-07-01

**Authors:** Ajoy Kumer, Unesco Chakma

**Affiliations:** a Department of Chemistry, European University of Bangladesh, Gabtoli, Dhaka, 1216, Bangladesh; b Department of Chemistry, Bangladesh University of Engineering Technology, Dhaka, 1000, Bangladesh; c Department Electrical and Electronics Engineering, European University of Bangladesh, Gabtoli, Dhaka, 1216, Bangladesh

**Keywords:** Band gap, Density of states, Photocatalyst, Dielectric function

## Abstract

For developing the stannite type quarterly crystal photocatalyst, the electronic structure and optical properties of ZnAg_2_GeSe_4_, ZnAg_2_Ge_0.93_Fe_0.07_Se_4_ and ZnAg_2_Ge_0.86_Fe_0.14_Se_4_ were calculated and compared with the parent stannite type quarterly crystal, ZnAg_2_GeS_4_. First of all, the four functionals, such as GGA with PBE, GGA with RPBE, GGA with WC and LDA with CA-PZ functionals were used for primary screening of electronic band structure and structural geometry for ZnAg_2_GeS_4_ while the band gap was in 0.93, 0.97, 0.77 and 0.67 eV, respectively. It must be mentioned that the experimental value of ZnAg_2_GeS_4_ was 0.94 eV so that the GGA with PBE showed the overlapping value of band gap. The main focus of this paper is to evaluate the band structure of newly predicted the stannite type quarterly crystal, ZnAg_2_GeSe_4_ using four methods replacing the Sulfur atom by Serium atom on ZnAg_2_GeS_4_. The band gap for four methods, such as GGA with PBE, GGA with RPBE, GGA with WC and LDA with CA-PZ functionals, were calculated in 0.84 eV, 0.92 eV, 0.68 eV and 0.58 eV. Afterward, Fe atom was doped by two portions, like 7% and 14%, to make the empirical formula, ZnAg_2_Ge_0.93_Fe_0.07_Se_4_ and ZnAg_2_Ge_0.86_Fe_0.14_Se_4_. The numerical values of band gaps for ZnAg_2_Ge_0.93_Fe_0.07_Se_4_ and ZnAg_2_Ge_0.86_Fe_0.14_Se_4_ were 0.43 eV, 0.53 eV, 0.35 eV and 0.18 eV and 0.24 eV, 0.31 eV, 0.18 eV and 0.08 eV, respectively, using the four respected DFT methods. For their contributed orbitals of each atom on crystal, the density of state and the partial density of state for ZnAg_2_GeSe_4_, ZnAg_2_Ge_0.93_Fe_0.07_Se_4_ and ZnAg_2_Ge_0.86_Fe_0.14_Se_4_ crystals were simulated through the GGA with PBE method as standard regarding the calculation of band gap study comparison with experimental magnitude. For giving the further information about the nature in case of optical evidence, the six optical properties, such as absorption, reflection, refractive index, conductivity, dielectric function and loss function were calculated, and make a comparative study. In case of UV light absorption in lighten to optical parameters, the ZnAg_2_Ge_0.86_Fe_0.14_Se_4_ can show the highest absorption up to convenience energy region as photocatalyst.

## 1 Introduction

Photocatalyst technology has considered as the most spreading and sustainable methods due to various facilities for the waste water treatment. The wastes, which have been produced from numerous industries, such as textile, readymade garments, pharmaceuticals, paint industries, and household wastes, are alarming for our human being in the present times. Particularly, these wastes make the high-end warming and threatening for developed and developing countries in our globe for both of aquatic and non aquatic environment whereas it must be reported that more than 30% natural environment had been destroyed last five decades [1, 2]. First of all, the annual water marks was found to be 1.8 billion m^3^ for only textile industry, and other industries consumed more than this amounts. The total amount of water was taken from the ground water which may result in depletion of groundwater level, other side it can lead to major health problems for the local people. Secondly, in case of domestic sewage, about 13468 minimum liquid discharge (MLD) of wastewater has been generated by overall industries, and 60% of that is treated for removal the toxic chemicals. Laila Hossain et al 2018 reported that about 23% fresh water will be reduced due to only textile industries water consumption in Bangladesh although Bangladesh is considered the 2^nd^ largest exporter country in our globe [3]. The other rationale is noted for waste water from textile industries that the organic dyes and pigments with polymers or fabrics are the vital contributor for producing such waste, and all of these are highly toxic for both aquatic and non aquatic living organisms. Moreover, the levels of ground water will be declined day by day for towering uses of water in households, industries and agricultural sectors that give us alarming point for our survival. In this case, the recycling or reuse of water is one alternative way to save and maintaining the sustainability of our environment where the photocatalyst and photocatalysis is the top most choosing avenue for researcher for its multifarious effects and facilities. Beginning with, a photocatalyst can be used for the purification of wastewater containing organic pollutants own to an advanced green and sustainable oxidation process [4], low cost, easy to handling, high stability and relatively low toxicity [5, 6, 7, 8, 9, 10]. To say more about its advantages, its reaction system is not high complex, short processing time, reusable and recyclable, non consumption of oxygen, and self regenerated and high level of UV or sun light absorption. Regarding these cases, photocatalytic technology has gained much popularity in recent years as well a user-friendly technology.

Though the current technology hardly meets the industrial requirement totally for many limitations, the most difficult task in this technology is to come up with an ideal photocatalyst having the four basic features, such as high photocatalytic efficiency [11], a wide specific surface area [12], the best utilization of sunlight (lower than 10% of solar radiation for TiO_2_), and recyclability [13]. Semiconductor-based photocatalysts have been admiring an effective alternative sources of the abundance of solar energy for dye and pigment degradation [14]. The basic process of photocatalysis consists of generating photo-induced electron-hole pairs [15] that lead to superoxide free radicals and hydroxyl free radicals by reacting with oxygen and water molecules and constitutes the characteristic active species causing organic pollutant photodegradation [16]. Researchers have been searching the substitute of the most commercial photocatalysts, such as SnO_2_, WO_3_, TiO_2_, CeO_2_ and ZnO [17, 18] which are the most capable to absorb the high level of UV light absorption and show the highest level of efficiency. However, this study has been designed to provide the new finding of photocatalysts insights into theory on basis of band gap concept.

There are lots of synthesis and experimental studies on Ag_2_BCX_4_ (B=Zn, Cd, Hg, Pb, Fe, Mn, C=Si, Ge, Sn; X = S, Se, Te) as the stannite type quarterly crystal [19, 20, 21]. Many metals sulfide photocatalysts show the activity using hydrogen progression under visible light irradiation having the occurrence of a sacrificial electron donor, which has the absorption ability of lower range from 400 to 430 nm [22]. Mykola Moroz et al. 2019 [23] reported the value of band gap was 2.2 eV of Ag_2_ZnGeS_4,_ which did not utilize all range of visible light due to its wide band gap. Afterward, the Cu_2_ZnSnS_4_ has recognized and reported as abundant and environmentally friendly photocatalyst materials, as well as perovskite solar cell, photovoltaic applications [24], has direct band gap of 1.5 eV [25, 26, 27, 28]. Moreover, it has been considered as a candidate for solar energy conversion through both photovoltaic and photocatalysis. The stanning-type multi-component sulfide (Ag_2_ZnSnS_4_) was reported the band 2.01 eV and after 1.0 wt% Pt-loading, the Ag_2_ZnSnS_4_ could absorb the more than 15 % UV light from 6% UV light absorption [29], as well as Ni- or Pb-doped Ag_2_ZnSnS_4_ enhanced the CO_2_ reduction of artificial photosynthesis using a simple Z-scheme system [30]. During last two decades, there were developed lots of metals sulfides complexes, such as, Au_2_Cs_2_I_6_, Ag_2_GeBaS_4_, Ag_2_ZnSnS_4_, AgCuPO_4_ [21, 31], Ag_2_FeSnS_4_(Cu, Ag)_2_ZnSnS_4_ [31] and (Cu, Ag)_2_ZnSnSe_4_ solid solutions [20] for using as advanced photocatalyst, optoelectronics, semiconductor and solar cell. Due to having the noble applications in the area of photocatalysts and electronics, the new metals sulfide stannite type quarterly crystal has been designed by computational tools. Now, Ag_2_ZnSnS_4_ has selected as base crystal, and it has been converted in ZnAg_2_GeSe_4_, ZnAg_2_Ge_0.93_Fe_0.07_Se_4_ and ZnAg_2_Ge_0.86_Fe_0.14_Se_4_ to make a comparative study through the four DFT functionals, such as Generalized Gradient Approximation (GGA) with Perdew Burke Ernzerhof (PBE), Generalized Gradient Approximation (GGA) with Revised Perdew Burke Ernzerhof functional, (RPBE), Generalized Gradient Approximation (GGA) with Wilson-Levy (WL) and Local Density Approximation (LDA) with Ceperley and Alder and Perdew and Zunger (CA-PZ) functionals with both of their advantages and disadvantages by computational details. The Se atom will have to be new area as selenium metal complexes for photocatalyst. As the photocatalytic activity increases with decreasing band gap, band gap concept has selected under this study, and takes doping policy to fall up band gap. The reason to select the Se atom is the large surface area, which indicates the more reactive catalyst [32, 33, 34]. On the other hand, Fe doping is most key point for reducing band gap and enhances photocatalytic behavior [35].

## 2 Computational methods

**Figure 1:**
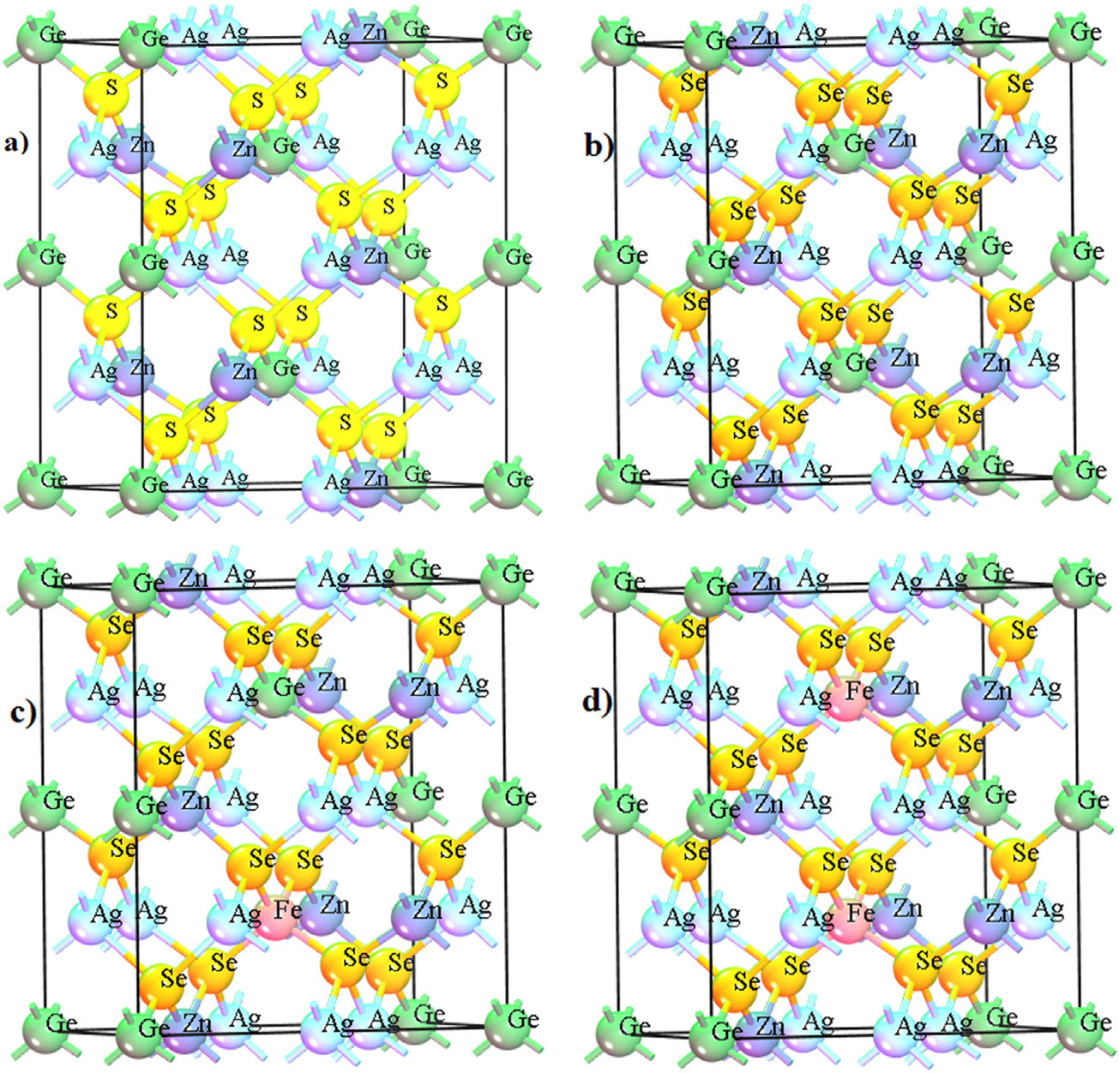
a) Optimized structure of ZnAg_2_GeS_4_, b) Optimized structure of ZnAg_2_GeSe_4_, c) Optimized structure of ZnAg_2_Ge_0_._93_Fe_0_._07_Se_4_, d) Optimized structure of ZnAg_2_Ge_0.86_Fe_0.14_Se_4_.

At first, the structural optimization for ZnAg_2_GeS_4_ crystal had performed by GGA with PBE method. It was illustrated that the tetragonal type, space group $\text{I}\bar{4}\left[82\right]$ was selected for computational study, which gave the similar value of experimental data shown in Table 1. For simulation, the convergence criterion for the force between atoms was 2 × 10^−6^ eV/atom, 1 × 10^−5^Å as the maximum displacement and 1 × 10^−5^ eV/atom as the total energy, and the same condition was applied for ZnAg_2_GeSe_4_, ZnAg_2_Ge_0.93_Fe_0.07_Se_4_ and ZnAg_2_Ge_0.86_Fe_0.14_Se_4_ keeping the cut off at 523, and k point at 4 × 4×2 with norm-conserving pseudopotentials functional. The 2 × 1×1 supercell models were considered to simulate the structural electronic and optical properties of ZnAg_2_GeS_4_, ZnAg_2_GeSe_4_, ZnAg_2_Ge_0.93_Fe_0.07_Se_4_ and ZnAg_2_Ge_0.86_Fe_0.14_Se_4_ shown in Figure 1(a), (b) and (c), respectively. First of all, the method of GGA with PBE was executed from CASTEP code from material studio 8.0 [36] for calculating the electronic structure for ZnAg_2_GeS_4_, ZnAg_2_GeSe_4_, ZnAg_2_Ge_0.93_Fe_0.07_Se_4_ and ZnAg_2_Ge_0.86_Fe_0.14_Se_4_ crystals. Then, density of states and optical properties were calculated with same condition. For the more investigation for calculating the band gap, by GGA with RPBE, GGA with WC and LDA with CA were demonstrated for all crystals in the same condition to make a comparative study.

**Table 1:** Structural calculation by four methods of ZnAg_2_GeS_4_, ZnAg_2_GeSe_4_, ZnAg_2_Ge_0.93_Fe_0.07_Se_4_ and ZnAg_2_Ge_0.86_Fe_0.14_Se_4_.

Methods	a	b	c	α	β	γ	Crystal type	Space group	Density
GGA, PBE	6.671Å	6.671Å	6.671Å	128.48°	128.48°	75.849°	tetragonal	$\text{I}\bar{4}$[82]	4.52 g/cm^3^
GGA, RPBE	6.671Å	6.671Å	6.671Å	128.48°	128.48°	75.849°	tetragonal	$\text{I}\bar{4}$[82]	4.52 g/cm^3^
GGA, WC	6.671Å	6.671Å	6.671Å	128.48°	128.48°	75.849°	tetragonal	$\text{I}\bar{4}$[82]	4.52 g/cm^3^
LDA,CA-PZ	6.671Å	6.671Å	6.671Å	128.48°	128.48°	75.849°	tetragonal	$\text{I}\bar{4}$[82]	4.52 g/cm^3^

## 3 Results and discussions

### 3.1 Optimized structure

The values of lattice parameter for ZnAg_2_GeS_4_, ZnAg_2_GeSe_4_, ZnAg_2_Ge_0.93_Fe_0.07_Se_4_ and ZnAg_2_Ge_0.86_Fe_0.14_Se_4_ were calculated from the materials studio after optimizing their crystal structures which had listed in Table 1 as the basic structural unit through the four methods, and try to keep its similar parameters getting a comparative study at a point. Withal, it must be noted for structural optimization showing in Figure 1(a)–(d) simulated by GGA with PBE which was considered as the standard functional of DFT to calculate the electronic structure and optical properties of crystal having heavy metal atoms.

### 3.2 Band structure

**Figure 2:**
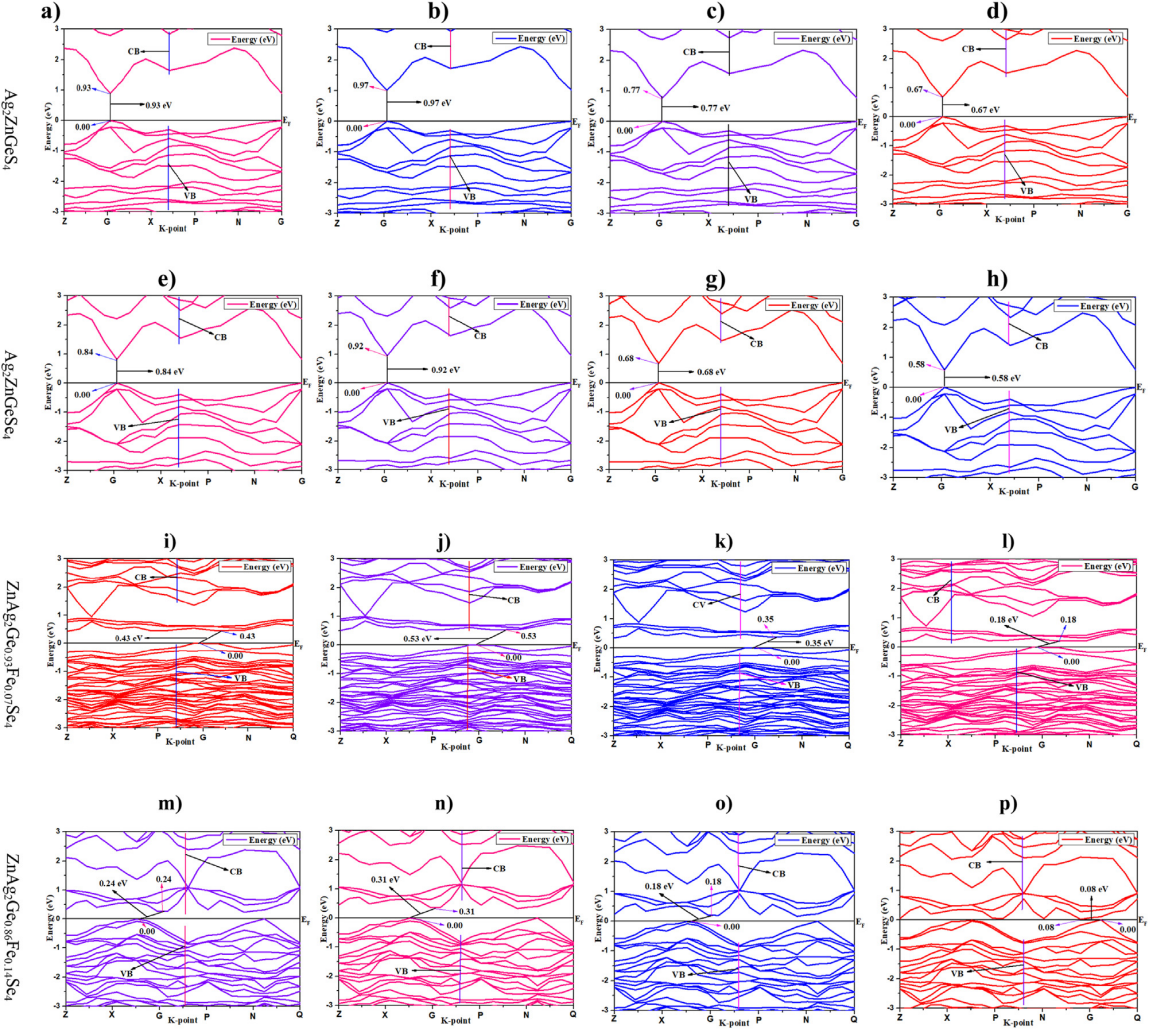
a) Band structure by GGA, PBE for Ag_2_ZnGeS_4,_ b) Band structure by GGA, RPBE for Ag_2_ZnGeS_4,_ c) Band structure by GGA, WC for Ag_2_ZnGeS_4_. d) Band structure by LDA, CA for Ag_2_ZnGeS_4_. e) Band structure by GGA, PBE for Ag_2_ZnGeSe_4_. f) Band structure by GGA, RPBE for Ag_2_ZnGeSe_4_. g) Band structure by GGA, WC for Ag_2_ZnGeSe_4_. h) Band structure by LDA, CA for Ag_2_ZnGeSe_4_. i) Band structure by GGA, PBE for ZnAg_2_Ge_0.93_Fe_0.07_Se_4_. j) Band structure by GGA, RPBE for ZnAg_2_Ge_0.93_Fe_0.07_Se_4_. k) Band structure by GGA, WC for ZnAg_2_Ge_0.93_Fe_0.07_Se_4_. l) Band structure by LDA, CA for ZnAg_2_Ge_0.93_Fe_0.07_Se_4_. m) Band structure by GGA, PBE for ZnAg_2_Ge_0.86_Fe_0.14_Se_4_. n) Band structure by GGA, RPBE for ZnAg_2_Ge_0.86_Fe_0.14_Se_4_. o) Band structure by GGA, WC for ZnAg_2_Ge_0.86_Fe_0.14_Se_4_. p) Band structure by LDA, CA-PZ for ZnAg_2_Ge_0.86_Fe_0.14_Se_4_.

The electronic band structure of semiconductor composes by a low energy valence band (VB) and a high energy conduction band (CB) while the forbidden band might be defined as a band gap between CB and VB. For calculating the band gap of ZnAg_2_GeS_4_, ZnAg_2_GeSe_4_, ZnAg_2_Ge_0.93_Fe_0.07_Se_4_ and ZnAg_2_Ge_0.86_Fe_0.14_Se_4_, the Fermi energy level was placed at zero energy level using four functionals of DFT from CASTAP by material studio 8.0 showing in Table 2. From Figure 2(a)–(h), it has observed that both the minimum of conduction bands (MCB) and the maximum of valence bands (MVB) for two crystals are obtained in the G symmetry point. Consequently, it can be called as the direct band gap for Ag_2_ZnGeS_4_ and Ag_2_ZnGeSe_4_. After Fe atom doping on Ag_2_ZnGeSe_4_, the band gap has reduced, but they have shown the indirect band gap in case of all functionals.

**Table 2:** Band gap with respect to various functionals of crystals.

Crystals/functional	GGA with PBE	GGA with RPBE	GGA with WC	LDA with CA	Ref
ZnAg_2_GeS_4_ (standard)	0.93 eV	0.97 eV	0.77 eV	0.67 eV	0.942 eV [8, 29]
Ag_2_ZnGeSe_4_	0.84 eV	0.92 eV	0.68 eV	0.58 eV	Newly predicted
ZnAg_2_Ge_0.93_Fe_0.07_Se_4_	0.43 eV	0.53 eV	0.35 eV	0.18 eV	Newly predicted
ZnAg_2_Ge_0.86_Fe_0.14_Se_4_	0.24 eV	0.31 eV	0.18 eV	0.08 eV	Newly predicted

In case of GGA with PBE, the material Ag_2_ZnGeS_4_ acquired direct band gap, and its value was in 0.93 eV shown in Figure 2(a) which was similar to its experimental value (0.94 eV). Due to have accurate result using GGA with PBE, the band gap noticed at 0.84 eV from Figure 2(e) as a direct band gap material for Ag_2_ZnGeSe_4_. Figure 2(i) demonstrated that the band gap was at 0.43 eV for ZnAg_2_Ge_0.93_Fe_0.07_Se_4_, and this band gap was noticed as an indirect band gap. Moreover, the material, ZnAg_2_Ge_0.86_Fe_0.14_Se_4_, obtained the indirect band gap by 0.24 eV from Figure 2(m).

Secondly, for functional of GGA with RPBE, the band gap for Ag_2_ZnGeS_4_ and ZnAg_2_GeSe_4_ was recorded at 0.97 eV, 0.92 eV, shown in Figure 2(b) and (f), and it was considered as a direct band gap material. After Fe doped by 7 % and 14% replacing Ge atom with the molecular formula ZnAg_2_Ge_0.93_Fe_0.07_Se_4_ and ZnAg_2_Ge_0.86_Fe_0.14_Se_4,_ the band gap has found at 0.53 eV and 0.31 eV, respectively, with an indirect band gap from figure.

Furthermore, it had also justified by GGA with the WC, the band gap for Ag_2_ZnGeS_4_, Ag_2_ZnGeSe_4,_ ZnAg_2_Ge_0.93_Fe_0.07_Se_4_ and ZnAg_2_Ge_0.86_Fe_0.14_Se_4_ were 0.77 eV, 0.68 eV, 0.35 eV and 0.18 eV, respectively. Finally, LDA with CA was employed for Ag_2_ZnGeS_4_, Ag_2_ZnGeSe_4_ ZnAg_2_Ge_0.93_Fe_0.07_Se_4_ and ZnAg_2_Ge_0.86_Fe_0.14_Se_4_ in similar conditions, the band gap was recorded at 0.67 eV, 0.58eV, 0.18eV and 0.08eV, respectively.

On the other hand, to make a comparative study among employing the four methods, such as GGA with PBE, GGA with RPBE, GGA with WC and LDA with CA on same geometry of Ag_2_ZnGeS_4_, Ag_2_ZnGeSe_4,_ ZnAg_2_Ge_0.93_Fe_0.07_Se_4_ and ZnAg_2_Ge_0.86_Fe_0.14_Se_4,_ the calculated band structures were selected. It was observed that the similar fluctuation was illustrated between MCB and MVB although there were a small difference for the methods, GGA with WC and LDA with CA. The most noticeable change was found after Fe doping in type of band structure that the Ag_2_ZnGeS_4_ and Ag_2_ZnGeSe_4_ showed the direct band gap which are converted in indirect band gap for ZnAg_2_Ge_0.93_Fe_0.07_Se_4_ and ZnAg_2_Ge_0.86_Fe_0.14_Se_4_. In case of Ag_2_ZnGeS_4_, the functional GGA with PBE gave the almost similar value of band gap (0.93 eV) with experimental value (0.94 eV) which was accurate result comparison with their literature result of ZnAg_2_GeS_4_ from the materialproject.org, ID mp-1215558 [37]. The other three methods have showed a large deviation from experimental value. Consequently, it could be said that GGA with PBE has considered as the standard method for further calculation.

### 3.3 Density of state

**Figure 3:**
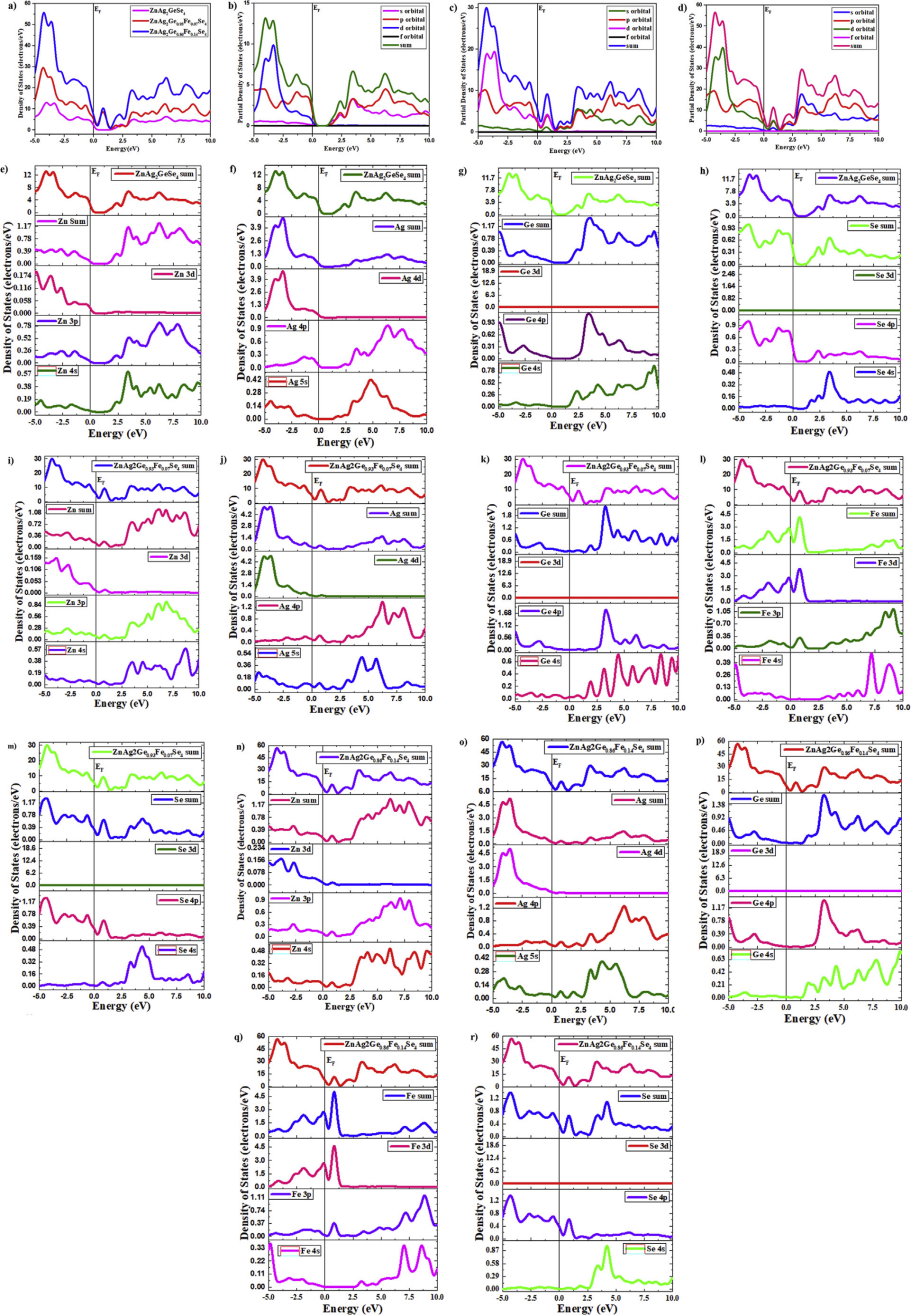
a) Total DOS for separate ato for ZnAg_2_GeSe_4_, ZnAg_2_Ge_0.93_Fe_0.07_Se_4_ and ZnAg_2_Ge_0.86_Fe_0.14_Se_4_, b) Partial Density of Statese for ZnAg_2_GeSe_4_, c) Partial Density of Statese for ZnAg_2_Ge_0.93_Fe_0.07_Se_4_, d) Partial Density of Statese for ZnAg_2_Ge_0.86_Fe_0.14_Se_4_, e) Zn Atom for ZnAg_2_GeSe_4_, f) Ag Atom for ZnAg_2_GeSe_4_, g) Ge Atom for ZnAg_2_GeSe_4_, h) Se Atom for ZnAg_2_GeSe_4_, i) Zn atom for ZnAg_2_Ge_0.93_Fe_0.07_Se_4_, j) Ag atom for ZnAg_2_Ge_0.93_Fe_0.07_Se_4_, k) Ge atom for ZnAg_2_Ge_0.93_Fe_0.07_Se_4_, l) Fe atom for ZnAg_2_Ge_0.93_Fe_0.07_Se_4_, m) Se atom for ZnAg_2_Ge_0.93_Fe_0.07_Se_4_, n) Zn atom for ZnAg_2_Ge_0.86_Fe_0.14_Se_4_, o) Ag atom for ZnAg_2_Ge_0.86_Fe_0.14_Se_4_, p) Ge atom for ZnAg_2_Ge_0.86_Fe_0.14_Se_4_, q) Fe atom for ZnAg_2_Ge_0.86_Fe_0.14_Se_4_, r) Se atom for ZnAg_2_Ge_0.86_Fe_0.14_Se_4_.

The density of states (DOS) and partial density of states (PDOS) express about the electronic contributions by orbital system which indicates how the electronic band structures have separated and produced by electrons. For ZnAg_2_GeSe_4_ ZnAg_2_Ge_0.93_Fe_0.07_Se_4_ and ZnAg_2_Ge_0.86_Fe_0.14_Se_4_ crystals, the total density of states (TDOS) composes by the elements (Ag, Zn, Ge, Fe and Se) which are measured and outlined in Figure 3(a)–(r), respectively. Moreover, the density of the state indicates the nature of electronic band structures and the splitting of an orbital. Actually, the DOS and PDOS are directly related the chemical reactivity descriptors, such as highest occupied molecular orbital (HOMO), lowest unoccupied molecular orbital (LUMO) and HOMO-LUMO gap, those three indicators are used to calculate ionization potential, electronegativity, hardness, softness and electron affinity of any crystals [38, 39]. HOMO is equal to the valence band while LUMO expresses to the conduction band. According to the mechanism of photocatalyst, the ⋅O^−2^ and ·OH free radicals react with the organic pollutants for degradation. Thus, the degradation completely depends on the production of ⋅O^−2^ and ·OH radicals. So it is the most urgent thing for photocatalyst that producing of ⋅O^−2^ and ·OH are the primary factor from H_2_O by adding catalysts.

The method of GGA with PBE was used to calculate the density of states (DOS) and partial density of states (PDOS) of Ag, Zn, Ge, Fe and Se elements for ZnAg_2_GeSe_4_, ZnAg_2_Ge_0.93_Fe_0.07_Se_4_ and ZnAg_2_Ge_0.86_Fe_0.14_Se_4_ crystals. There are a lot of literatures for calculating the DOS and PDOS from LDA to GGA method [40, 41]. Regarding this case, this function was used in this study.

From Figure 3(a), it is shown that the comparative contribution of ZnAg_2_GeSe_4_, ZnAg_2_Ge_0.93_Fe_0.07_Se_4_ and ZnAg_2_Ge_0.86_Fe_0.14_Se_4_ orbitals are responsible for VB and CB for which the DOS and PDOS are created. The TDOS of ZnAg_2_Ge_0.86_Fe_0.14_Se_4_ is much higher than TDOS of ZnAg_2_GeSe_4_ and ZnAg_2_Ge_0.93_Fe_0.07_Se_4_ in both VB and CB. From Figure 3(b), (c) and (d), it is found that the conduction band is mostly affected by p orbital, and valence band is also affected by d orbital for all of ZnAg_2_GeSe_4_, ZnAg_2_Ge_0.93_Fe_0.07_Se_4_ and ZnAg_2_Ge_0.86_Fe_0.14_Se_4_. Figure 3(e)–(r) show that the individual atom how can contribute to create the DOS and PDOS, while the orbital of Fe can significantly contribute to decline the band gap between VB and CB. The main cause for declining the DOS in the CB is explained as doping by Fe atom, and it is noted that Fe atom contributes in both valence band and conduction band. As a result, the level of valence band increases, as well as the level of the conduction band decreases. With respect to this causes, band gap has fallen down by doping Fe atom on Ag_2_ZnGeSe_4._ The study of TDOS for ZnAg_2_GeSe_4_ ZnAg_2_Ge_0.93_Fe_0.07_Se_4_ and ZnAg_2_Ge_0.86_Fe_0.14_Se_4_ crystals illustrates that the ZnAg_2_Ge_0.86_Fe_0.14_Se_4_ shows the highest electron density in both VB and CB than ZnAg_2_GeSe_4_ and it's in valence band is almost twice times. There is a small change in the conduction band, which is highly responsible for activity to conduct charge carriers of the free radical carrier as acting photocatalyst.

### 3.4 Photocatalytic activity

It is well known estimation that the photocatalytic reaction takes place by both oxidation and reduction processes due to active action of metal oxide or metal crystal as acting catalyst. In the case of oxidative reaction, the positive holes combine with directly water molecules from moisture, and produces hydroxyl radical although negative holes are responsible for reduction [42]. The mechanism is given below:

$$\begin{array}{ll} {\text{ZnAg}}_{2}{\text{GeSe}}_{4}/{\text{ZnAg}}_{2}{\text{Ge}}_{0.93}{\text{Fe}}_{0.07}{\text{Se}}_{4}/{\text{ZnAg}}_{2}{\text{Ge}}_{0.86}{\text{Fe}}_{0.14}{\text{Se}}_{4}+\text{hv}\to & {\text{ZnAg}}_{2}{\text{GeSe}}_{4}/{\text{ZnAg}}_{2}{\text{Ge}}_{0.93}{\text{Fe}}_{0.07}{\text{Se}}_{4}/{\text{ZnAg}}_{2}{\text{Ge}}_{0.86}{\text{Fe}}_{0.14}{\text{Se}}_{4}\left({\text{h}}^{+}\&{\text{e}}^{-}\right)\\ \text{Photocatalyts} & \text{hole } \end{array}$$

$$\begin{array}{ll} \text{Oxidative reactions due to photocatalytic effect:} & \text{Reductive reactions due to photocatalytic effect:}\\ {\text{h}}^{+}+{\text{H}}_{2}\text{O}\to {\text{H}}^{+}+\;\cdot \text{OH} & {\text{e}}^{-}+{\text{O}}_{2}\to \cdot {\text{O}}_{2}^{-}\\ 2{\text{h}}^{+}+2{\text{H}}_{2}\text{O}\to 2{\text{H}}^{+}+2{\text{H}}_{2}{\text{O}}_{2} & \cdot {\text{O}}_{2^{-}}+{\text{H}}_{2}\text{O }+\;{\text{H}}^{+}\to {\text{H}}_{2}{\text{O}}_{2}+{\text{O}}_{2}\\ {\text{H}}_{2}{\text{O}}_{2}\to 2\cdot \text{OH} & {\text{H}}_{2}{\text{O}}_{2}\to 2\cdot \text{OH} \end{array}$$

The key task of the photocatalyst is explained in the approaching the producing of hydroxyl free radicals that can be able to combine with organic pollution for making the degradation product of pollution. From the reaction, it is transparently illustrated that the photo catalysis depends on the production of hydroxyl radicals which is also related to absorption of UV. The absorption of UV light correlates with band gap. In case of the most used photocatalysts, the band gap was found in the range of 3.2 to 2.8 eV or below, which corresponding to 387.45 nm–442.80 nm wavelength. In addition, it was quoted that the band gap was 1.8 eV or below 1.8 eV considered as good photocatalyst which was corresponded to 688.80 nm wavelengths. In our studies, the band gap for ZnAg_2_GeSe_4,_ ZnAg_2_Ge_0.93_Fe_0.07_Se_4_ and ZnAg_2_Ge_0.86_Fe_0.14_Se_4_ were recorded at 0.84 eV, 0.43 eV and 0.24eV, respectively. Among of them, ZnAg_2_Ge_0.93_Fe_0.07_Se_4_ and ZnAg_2_Ge_0.86_Fe_0.14_Se_4_ might be said as good photocatalyst which can be able to absorb the large range of UV light.

### 3.5 Optical properties

The action of photocatalyst conveys on the absorption ability of light, charge transportation and several active sites which are related to the magnitude of band gap and electrons or holes mobility in regarding of conductivity, reflectivity and refractive index. Next, the number of active sites with a large surface area of the molecule play a crucial role to absorb the pollutant because the greater surface area can produce the large number of active surface sites, thus increasing their decomposition or oxidation process.

#### 3.5.1 Optical reflectivity

**Figure 4:**
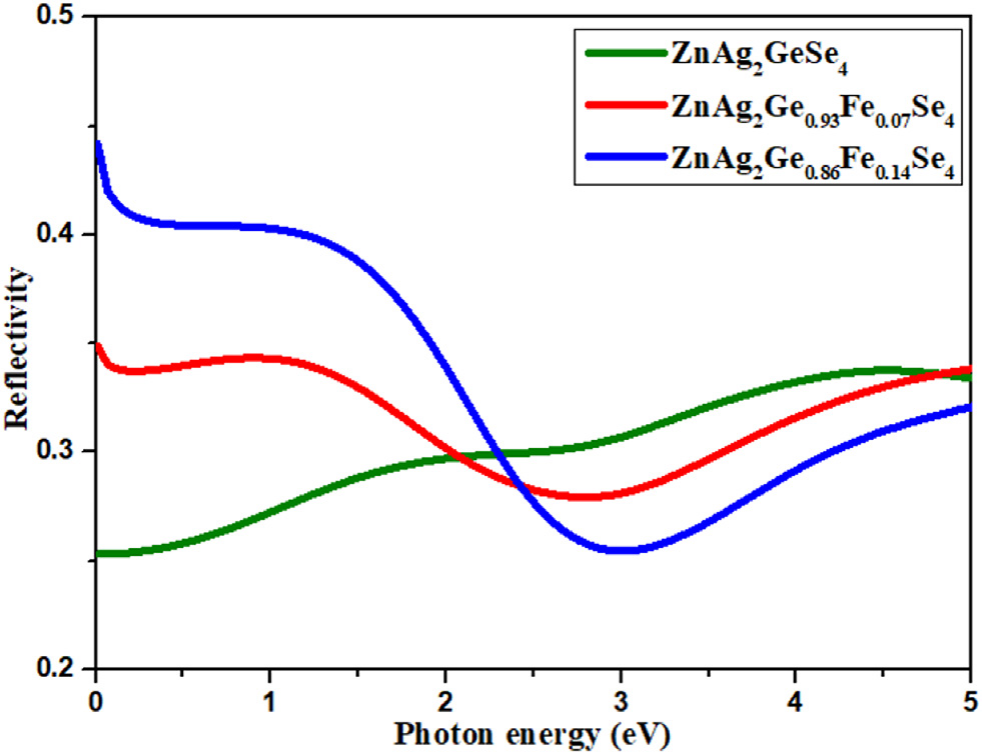
Reflectivity.

As a part of a number of repeated computational explorations of optical phenomenon, at first, it is directed related to the dielectric constant of ionic materials. The amount of light, that is incident on the surface of the photocatalytic materials, can be estimated from the reflectivity data and it is related to the absorbance of that material. There are a number of previous investigation that the lower reflectivity indicates the higher UV or visible light absorption [43]. In this exploration, the reflectivity of ZnAg_2_GeSe_4,_ ZnAg_2_Ge_0.93_Fe_0.07_Se_4_ and ZnAg_2_Ge_0.86_Fe_0.14_Se_4_ have recorded with the range of energy 0 eV–5 eV. With the starting, the reflectivity of ZnAg_2_Ge_0.86_Fe_0.14_Se_4_ has about 0.45 while the reflectivity of ZnAg_2_Ge_0.93_Fe_0.07_Se_4_ and ZnAg_2_GeSe_4_ were 0.35 and 0.25. With increasing photon energy, the reflectivity of ZnAg_2_GeSe_4_ has gradually increased to reach in 0.32 while the reflectivity of ZnAg_2_Ge_0.93_Fe_0.07_Se_4_ and ZnAg_2_Ge_0.86_Fe_0.14_Se_4_ have decreased up to energy 2.5 eV then, it has increased and reached in similar to ZnAg_2_GeSe_4_ (see Figure 4).

#### 3.5.2 Absorption

**Figure 5:**
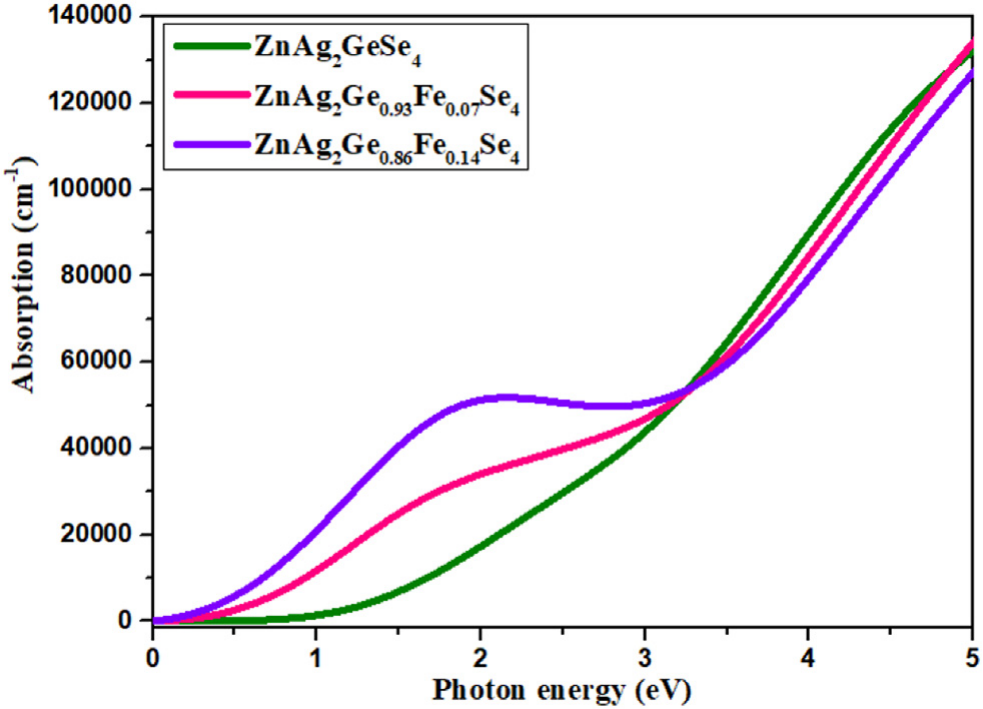
Absorption.

The polycrystalline polarization method is utilized to calculate the optical absorbance of the ZnAg_2_GeSe_4_, ZnAg_2_Ge_0.93_Fe_0.07_Se_4_ and ZnAg_2_Ge_0.86_Fe_0.14_Se_4_ materials, and the method comprises of the electric field vector as an isotropic average over all directions. The obtained absorbance peaks, depicted in Figure 5, is attributed to the photo transition energies from the maximum valence band (MVB) to the minimum conduction band (MCB) under visible light irradiation, which indicates that this material can absorb photons of visible range. From the initiation photon energy, the absorption of ZnAg_2_Ge_0.86_Fe_0.14_Se_4_ is much higher among ZnAg_2_GeSe_4_ and ZnAg_2_Ge_0.93_Fe_0.07_Se_4,_ and it is gradually maintained up to photon energy 3.0 eV. Afterward, it is noted that the absorption in near to 1.0 eV of ZnAg_2_Ge_0.86_Fe_0.14_Se_4_ is about five times greater than other two crystals, and at energy 3.3 eV, all of three crystals meet in a point. After then, opposite trends are obtained.

#### 3.5.3 Refractive index

**Figure 6:**
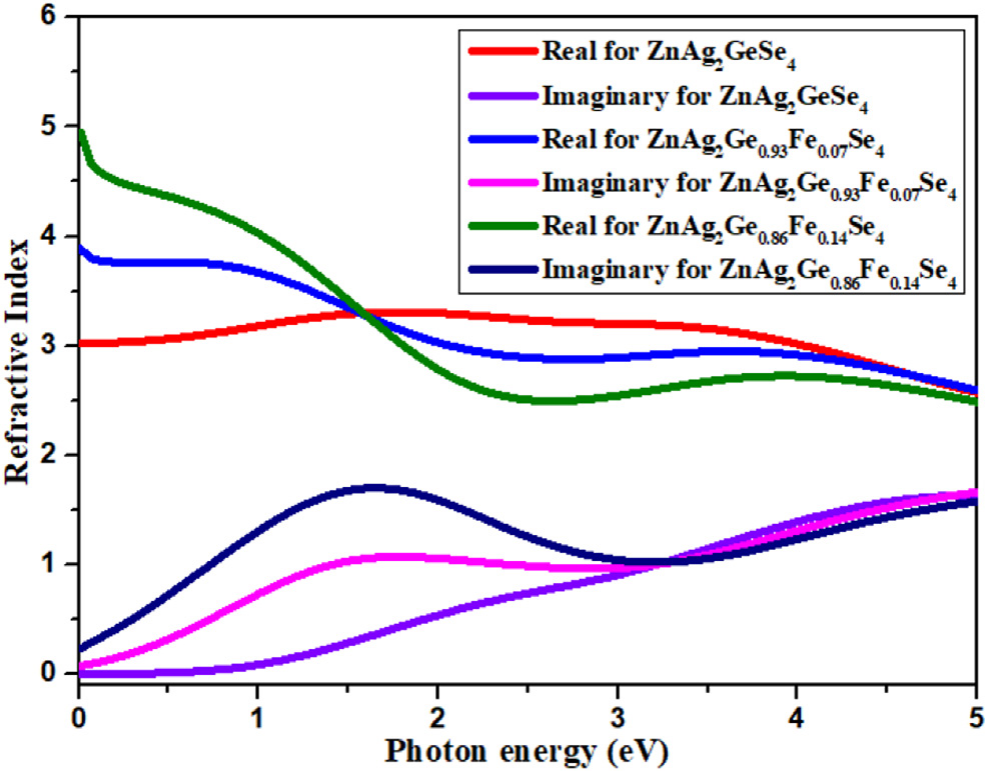
Refractive index.

The refractive index of a material is an impactful parameter for measuring the photon absorption throughout the process of chemicals degradation from the solutions. A large value of the refractive index is associated with the greater denser medium, which was reported in a previous investigation [44]. Figure 6 displays the refractive index as a function of photon energy where the red, blue and olive lines represent the real part of ZnAg_2_GeSe_4_, ZnAg_2_Ge_0.93_Fe_0.07_Se_4_ and ZnAg_2_Ge_0.86_Fe_0.14_Se_4_ respectively, and the navy blue, magenta and light violet lines illustrate for the imaginary part, and both the parts have an inverse pattern. At the initial point of photon energy, the refractive index is higher for the real part while the imaginary part stays almost closed to zero. After that, the magnitude of real part starts to decline and reached the lowest point, but on the contrary, the imaginary part rises gradually and achieves the highest point at around the photon energy of 1 eV. After doping, the real part sharply falls down at 2 eV photon energy, then it becomes constant, and the imaginary part is almost similar as compared to undoped.

#### 3.5.4 Dielectric function

The dielectric function is the basis function for calculating some optical properties, such as reflectivity, refractive index which are recountd to adsorption as the following equation for solid material [45].

$$\text{ε }=\;{\text{ε}}_{1}\left(\text{ω}\right)+{\text{iε}}_{2}\left(\text{ω}\right)$$

Here, $\varepsilon_{1}$ (ω) indicates the real part of the dielectric constant, and ${\text{ε}}_{2}\left(\text{ω}\right)$ stand for the dielectric loss factor (imaginary part). A dielectric function is the molecular properties related to the space of materials that belongs to physically equivalent to the permittivity or absolute permittivity. The energy storage potential in the electric field is maintained by real part of the dielectric constant and imaginary part indicates the opposite even for electric potential energy.

**Figure 7:**
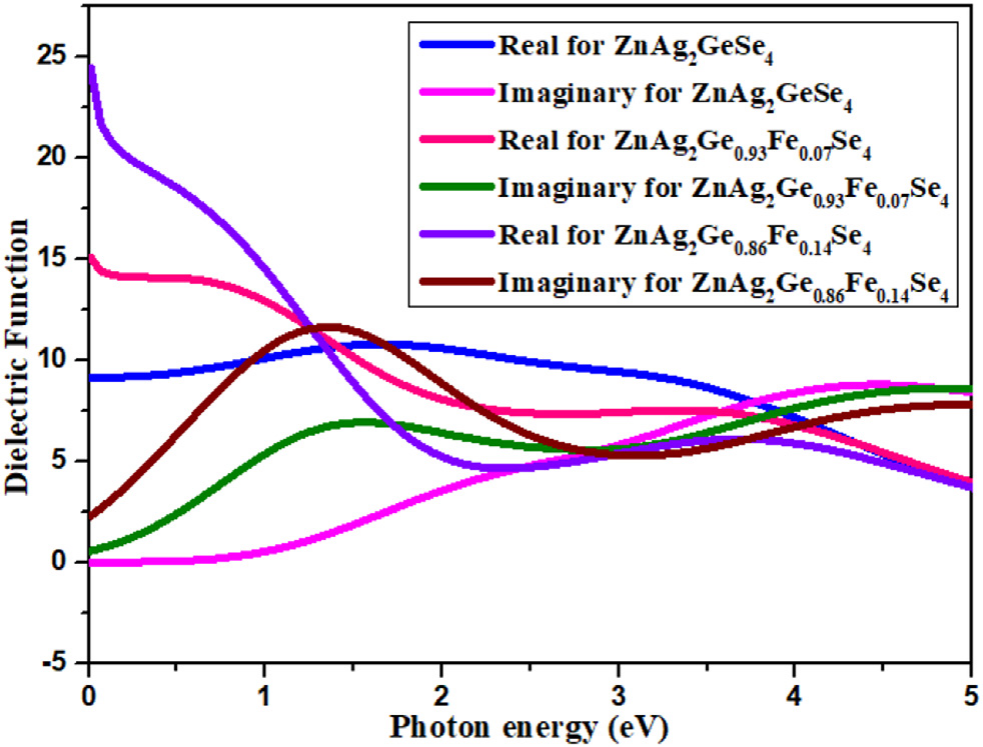
Dielectric function.

From Figure 7, the real part always remains the higher portions than the imaginary part is less from 0 eV to 3 eV frequencies, but from 3.5 eV to 5 eV, the imaginary part is larger than the real part. There have a significant change at initial photon energy whereas the 14% doping is found in peak point for real part, and 7% is in second position. In case of imaginary part, undoped is at zero point while 14% doping is at 0.25 for initial energy.

#### 3.5.5 Conductivity

**Figure 8:**
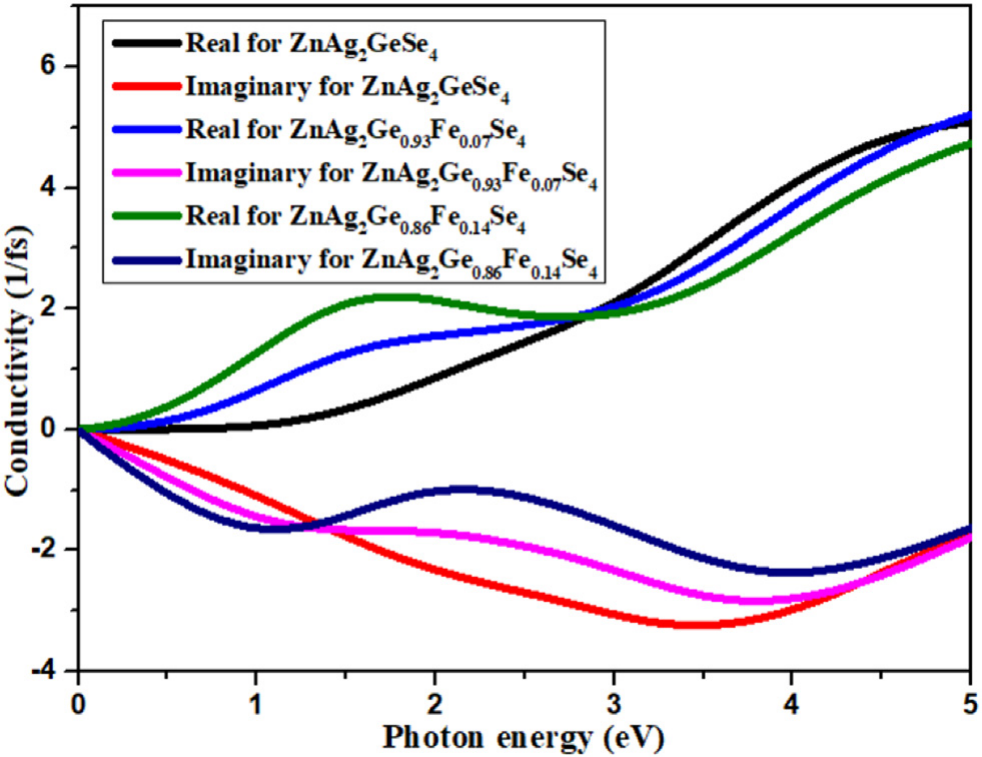
Conductivity.

The optical conductivity conveys a relation induced current density and electric field in term of energy band of electrons available in valence band conduction band. This means that these transporting electrons are produced for having loose holes and free electrons in the crystal materials. To become an active photocatalyst, the band gap between CB and VB is less than 1.8 eV. From Figure 8, the conductivity for doped and undoped is at about 0.0 eV for both the real and imaginary part, which is almost overlapping for good photocatalyst. In the band gap of 0.84 eV and its corresponding photon energy 0.84 eV, the conductivity for real part for ZnAg_2_Ge_0.86_Fe_0.14_Se_4_ has recorded as the highest point and ZnAg_2_Ge_0.93_Fe_0.07_Se_4_ is in second position. At 2.72 eV photon energy, the conductivity for real part is almost same point and afterward it shows opposite trends. Moreover, Inverse trend has found for the imaginary part for conductivity.

#### 3.5.6 Loss function

**Figure 9:**
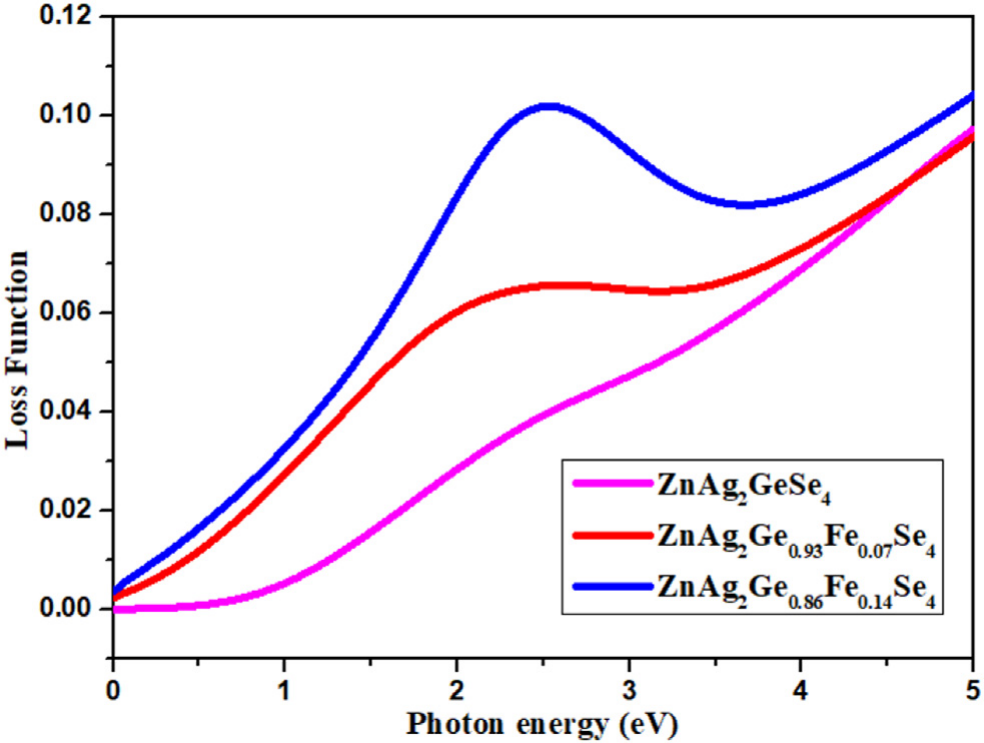
Loss function.

The loss function is composed by two regions of electronic photon energy parts, such as high photon energy part and low photon energy part, for optical properties. The ionization edge, indicating the oxidation state of d orbital splitting for metals of center atom in complex compounds, remains the range at 2.5 eV or more than 2.5 eV in shown Figure 9. It is true that the d orbital splitting for ZnAg_2_Ge_0.86_Fe_0.14_Se is achieved the maximum loss function at point 2.5 eV, and ZnAg_2_Ge_0.93_Fe_0.07_Se_4_ conveys the second higher maximum point which is caused due to Fe atom doping into the crystal, ZnAg_2_GeSe_4._

## 4 Conclusion

To put it briefly, using first principle method for calculation of electronic structure and optical properties, the ZnAg_2_GeSe_4_, ZnAg_2_Ge_0.93_Fe_0.07_Se_4_ and ZnAg_2_Ge_0.86_Fe_0.14_Se_4_ solid crystals have screened and investigated. According to their band gap, the ZnAg_2_GeSe_4_ is a semiconductor material having band gap 0.93 eV which is experimental value. For calculating the electronic structure, the GGA with PBE functional has considered the suitable method among the other three methods, such as GGA with RPBE, GGA with WC and LDA with CA-PZ functional for calculating the electronic structure of heavy metal containing crystals. Moreover, as lower band gap (less than 1.8 eV) can absorb about 688.80 nm wavelength, it is not sufficient for acting better photocatalyst because more narrow or small band gap has to require for UV light absorption having larger wavelength 688.80 nm. In this study, the ZnAg_2_GeSe_4_ crystal has designed for theoretical investigation in term of band gap concept, DOS, PDOS, optical properties and Fe doping activity where the band gap has obtained by 0.84 eV, 0.43 eV and 0.24 eV for ZnAg_2_GeSe_4_, ZnAg_2_Ge_0.93_Fe_0.07_Se_4_ and ZnAg_2_Ge_0.86_Fe_0.14_Se_4,_ respectively by the method of GGA with PBE functional. So that it could as well be said that the band gap has reduced after doping and the photocatalytic activity has increased. Furthermore, the DOS and PDOS show how all atoms contribute on band structure in VB and CB, as well as absorption capacity. Secondly, doping of Fe atom in crystal the significance in electronic band structure and optical properties have illustrated and have to say that Fe atom has a enormous contribution for reducing the band gap regarding to enhance the UV light absorption. Finally, the ZnAg_2_Ge_0.86_Fe_0.14_Se_4_ could be concreted to be stronger among ZnAg_2_GeSe_4_ and ZnAg_2_Ge_0.93_Fe_0.07_Se_4_ as photocatalyst.

## Declarations

### Author contribution statement

Ajoy Kumer: Conceived and designed the experiments; Analyzed and interpreted the data; Wrote the paper.

Unesco Chakma: Performed the experiments; Contributed reagents, materials, analysis tools or data.

### Funding statement

This research did not receive any specific grant from funding agencies in the public, commercial, or not-for-profit sectors.

### Data availability statement

No data was used for the research described in the article.

### Declaration of interests statement

The authors declare no conflict of interest.

### Additional information

No additional information is available for this paper.
